# Primary ligament sutures as a treatment option of knee dislocations: a meta-analysis

**DOI:** 10.1007/s00167-012-2154-8

**Published:** 2012-08-07

**Authors:** Karl-Heinz Frosch, Achim Preiss, Saskia Heider, Dirk Stengel, Peter Wohlmuth, Martin F. Hoffmann, Helmut Lill

**Affiliations:** 1 Department of Trauma and Reconstructive Surgery, Asklepios Clinic St. Georg, Lohmühlenstr. 5, 20099 Hamburg, Germany; 2 Department of Trauma Surgery, Plastic and Reconstructive Surgery, Georg-August-University, Goettingen, Germany; 3 Departments of Trauma and Orthopaedic Surgery, Unfallkrankenhaus Berlin and University Hospital of Greifswald, Berlin, Germany; 4 Proresearch, Asklepios Clinic St. Georg, Hamburg, Germany; 5 Clinic of Trauma and Reconstructive Surgery, Diakoniekrankenhaus Friederikenstift, Hannover, Germany

**Keywords:** Knee, Knee dislocation, Ligament, Reconstruction, Suture

## Abstract

**Purpose:**

Treatment of knee dislocation is still controversial. There is no evidence to favour ligament suture or reconstruction. Until now, no meta-analyses have examined suture versus reconstruction of cruciate ligaments in knee dislocations with respect to injury pattern and rupture classification.

**Methods:**

We searched Medline, the Cochrane Controlled Trial Database, and EMBASE for studies on surgical treatment for ‘knee dislocation’ and ‘multiple ligament injured knee’. A meta-analysis was performed using individual patient data.

**Results:**

Nine studies including 195 patients (200 knees) with a mean age of 31.4 (±13) years fulfilled the study requirements. Thirteen cases of type II dislocations, 63 cases of type III medial, 84 cases of type III lateral, and 40 cases of type IV dislocations, according to Schenck’s classification, were found. Poor or moderate results were found in 70 % of patients without surgical treatment of ACL or PCL (*n* = 27). Patients (*n* = 40) treated by sutures of the ACL and PCL demonstrated a significantly greater proportion of excellent or good results (40 and 37.5 %, respectively) (*p* < 0.001). Patients who underwent reconstruction of the ACL and PCL (*n* = 75) showed excellent or good results (28 and 45 %, respectively). No significant difference was found when comparing suture versus reconstruction of the ACL and PCL (n.s.). The outcome depends considerably on Schenck’s injury pattern classification.

**Conclusion:**

Conservative treatment after knee dislocation yields poor clinical results. Suture repair of cruciate ligaments can still serve as an alternative option for multiligament injuries of the knee and achieve good clinical results, which are comparable to those of ligament reconstruction. The data provided by this meta-analysis should be reinforced by a prospective study, in which suture repair and ligament reconstruction are compared.

**Level of evidence:**

IV.

## Introduction

Knee joint dislocation is rare and accounts for only approximately 0.02 % of all musculoskeletal injuries [20]. Most published studies include only a small number of cases. Therefore, evidence-based treatment guidelines are lacking and treatment options are controversial. The incongruence of study populations can lead to difficulties when comparing studies. Because of the lack of homogeneity in the injury pattern after knee dislocations, multiple therapeutic regimens have been recommended [1, 3, 6, 10, 16, 18, 21, 27, 28, 32]. Non-operative treatment of knee dislocation leads to poor short- and long-term outcomes and is no longer recommended [8, 13]. Two-stage management of multiple knee ligament injuries is widely accepted [4, 33]. According to the 2-stage regimen, medial and/or lateral collateral ligament sutures are performed within 8–10 days of injury, followed by reconstruction of the anterior and/or posterior cruciate ligaments after 6–8 weeks [33]. Good clinical outcome has been demonstrated in 70 % of patients undergoing a 2-stage procedure [22]. A meta-analysis by Levy et al. [11] found significantly better results in patients undergoing autologous tendon reconstruction of cruciate ligaments compared with patients undergoing suture repair.

The major problems of most published studies on knee joint dislocations are as follows: (1) no sufficient differentiation between acute and chronic injuries and (2) the lack of correlation of clinical outcome with injury pattern and surgical treatment. Some studies perform suture repairs of the collateral ligaments without reconstruction. Others perform additional sutures of only one cruciate ligament [4, 20]. Existing meta-analyses and systematic reviews did not perform individual analyses [11] or account for injury patterns. Moreover, specifications about which ligaments have been treated by sutures are absent [11]. No information can be found about untreated isolated structures (e.g. ACL or PCL).

This study was initiated based on the lack of homogeneity in the most recent literature. Our goal was to provide more detailed information about treatment options for knee dislocations based on a meta-analysis of clinical outcome with respect to injury pattern and performed treatment. In particular, this study focused on the clinical results of anatomical suture repair of cruciate ligaments in knee joint dislocation versus reconstruction of the ACL and PCL.

## Materials and methods

A search of Medline, the Cochrane Controlled Trial Database, and EMBASE for studies on surgical treatment for ‘knee dislocation’ and ‘multiple ligament injured knee’ was performed in April 2009. Additionally, the reference list of each article was searched for additional studies. All studies providing individual patient data-specific injury classification, detailed treatment protocol, and follow-up examination were included.

The data collected for each patient included the following: age in years; gender; mechanism of injury (motor vehicle accident, accident, sports); number of ligaments ruptured (2–4 or the Schenck’s classification [23]); time between rupture and treatment; surgical or conservative treatment; single or multiple operations; and the occurrence of vascular and/or nerve injuries.

To provide as homogeneous data as possible all collected patient data were grouped according to the Schenck classification [23].

Articles were excluded when the injury pattern and treatment regimen of each individual patient was not exactly described and only pooled for mean values. Further exclusion criteria included the following: the lack of a treatment description for each injured ligament complex; ruptures of only one cruciate ligament, even if combined posterolateral or anteromedial instabilities were described; isolated medial or lateral instabilities with intact cruciates; no clinical findings of each patient presented; or if the latest clinical follow-up occurred less than 1 year ago. Patients without an assigned Lysholm or IKDC Score were also not included in the study.

The term ‘reconstruction’ was used in all articles for ACL and/or PCL replacement. Therefore, we used the term ‘reconstruction’ for ligament replacement in the present study.

Procedure success was rated as ‘excellent’, ‘good’, ‘fair’ and ‘poor’ according to IKDC values or, if unavailable, according to the Lysholm Score.

### Statistical analysis

Continuous data are reported as the means and standard deviations if the variables were normally distributed, or as medians, minima, first and third quartiles and maxima if they were not. Categorical data were described with absolute and relative frequencies.

Demographics, injury pattern according to Schenck and interventional data (‘suture’ or ‘reconstruction’) were associated with treatment success, an ordinal variable characterised as ‘excellent’, ‘good’, ‘fair’ or ‘poor’. Univariate Proportional Odds Models were applied to assess the effects of the variables on treatment response. Treatment effects, defined as the impact of ‘ACL, PCL reconstruction’, ‘ACL, PCL suture’, ‘ACL reconstruction only’ or ‘PCL reconstruction only’ on the clinical outcome, were determined by univariate Proportional Odds Models.

A multiple Proportional Odds Model was based on the only two studies [29, 34] with complete data. Backward-, forward- and stepwise-selection procedures were applied to the data.

Associations between the covariates and treatment response were described with odds ratios (OR) and 95 % confidence intervals of the OR point estimates.

All *p* values are two-sided, and *p* < 0.05 was considered significant. All calculations were performed with SAS statistical analysis software (SAS Institute Inc., version 9.2, Cary, NC, USA).

## Results

**Fig. 1:**
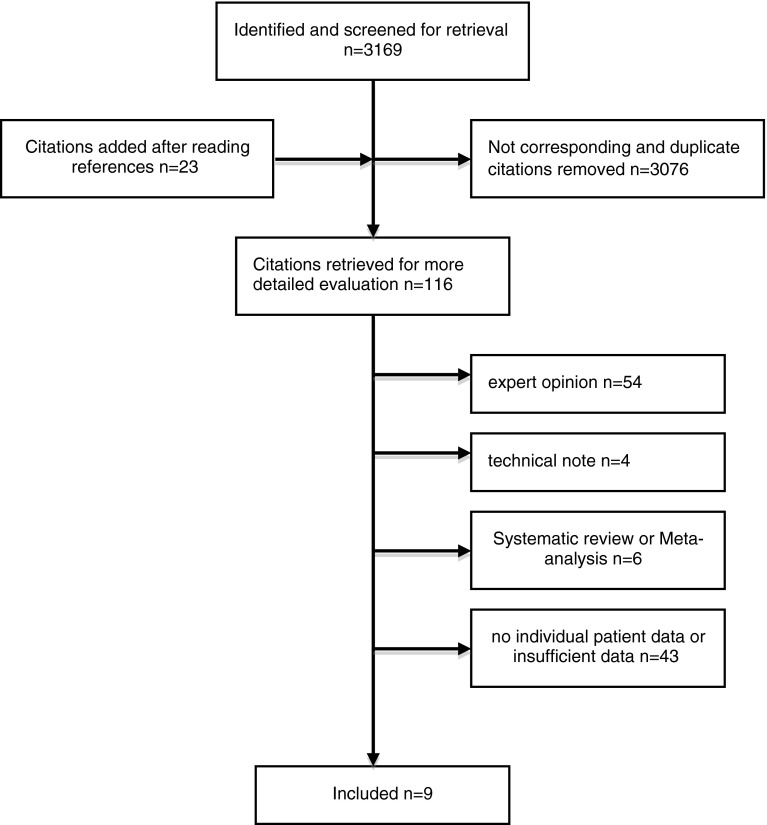
Study selection procedure

The inclusion criteria were met by eight articles and the national multicentre study of the German Society of Trauma Surgery (‘DGU’) [22] (Fig. 1). A total of 195 patients (200 knee joints) were included. Mean age was 31.4 (±13) years (Table 1). Time from trauma to surgery was 84.8 (±203.4) days. Suture repair was performed within 27 days after trauma.

**Table 1:** Patient data of the different studies and classification of knee dislocations according to Schenck et al. [23]

Author	Years	Level of evidence	Patients (knees)	Age (SD) in years	Follow-up (SD) in months	Classification according to Schenk (%)			
						Type II	Type III lateral	Type III medial	Type IV
Montgomery et al. [17]	1995	4	13 (13)	31.9 (9)	30.1 (21.9)	0 (0)	5 (38)	7 (54)	1 (8)
Montgomery et al. [17]	1995	4	12 (12)	37.4 (15)	83.7 (74.3)				
Shelbourne et al. [25]	2007	4	17 (17)	22.7 (5.4)	55.2	0 (0)	17 (100)	0 (0)	0 (0)
Owens et al. [18]	2007	4	25 (28)	35.0 (14)	48.0	1 (4)	16 (57)	1 (4)	10 (36)
Yeh et al. [34]	1999	4	23 (25)	37.8 (14.5)	27.4 (7.9)	0 (0)	9 (36)	12 (48)	4 (16)
Shapiro et al. [24]	1995	4	7 (7)	26.3	51.4				
Harner et al. [7]	2004	4	31 (31)	28.4 (10.9)	44.2 (15.4)	7 (23)	9 (29)	15 (48)	0 (0)
Washer et al. [31]	1999	4	13 (13)	27.5 (9.9)	38.4 (11.1)	0 (0)	6 (46)	7 (54)	0 (0)
Scheffler et al. [22]	2009	4	54 (54)	33 (14.3)	40.5 (23.3)	5 (9)	15 (28)	13 (24)	19 (35)
Bin et al. [4]	2007	4	14 (15)	31.2 (10.4)	88.9 (21.9)	0 (0)	5 (33)	7 (47)	3 (20)
Thomsen et al. [29]	1984	4	6 (6)	18.2 (1.2)	60 (41.6)	0 (0)	2 (33)	1 (17)	3 (50)
Total			215 (221)	31.4	47.0	13 (6)	84 (42)	63 (31)	40 (20)

**Fig. 2:**
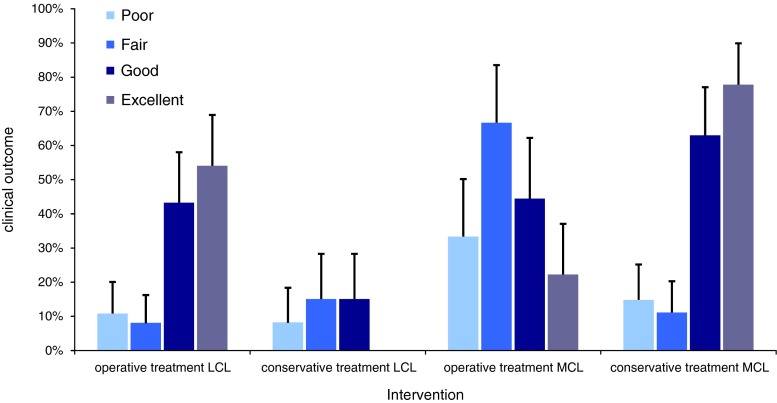
Mean values and standard deviations of grouped individual patient data are presented. Only patients with Type III and IV injuries were included. Clinical results depending on *MCL* (medial collateral ligament) and *LCL* (lateral collateral ligament) treatment. The grades of *excellent*, *good*, *fair* and *poor* were pooled as results from the Lysholm and/or IKDC Score. Operative treatment of the *LCL* led to significantly better results than conservative therapy. No significant difference between operative and conservative treatment of *MCL* could be detected

Thirteen cases of type II dislocation, 63 cases of type III medial (IIIM), 84 cases of type III lateral (IIIL), and 40 cases of type IV according to Schenck’s classification were found (Table 1). Type II injuries did not receive suture repair, in 9 cases ACL and PCL was reconstructed. Refixation or suture of the lateral coligament was performed in 53 cases (71 %, unknown *n* = 44) (Table 1). Seven patients (9.5 %) underwent ligament reconstruction. Non-operative treatment was performed in 14 cases (18.9 %). Injuries related to the medial collateral ligament were treated using suture or refixation techniques in 64 cases (80 %, unknown *n* = 20). Three patients (3.8 %) underwent replacement or augmentation of the medial collateral ligament. Non-operative treatment was performed in 13 cases (16.3 %) (Table 2). Compared with conservative therapy, repair of the posterolateral corner had a significant positive effect on the clinical result (*p* < 0.05), whereas MCL repair had no effect on the clinical outcome (n.s.) (Fig. 2).

**Table 2:** Different treatments of ACL (anterior cruciate ligament) and PCL (posterior cruciate ligament) in the evaluated studies

Author	Years	ACL			PCL		
		Suture (%)	Reconstruction (%)	No treatment (%)	Suture (%)	Reconstruction (%)	No treatment (%)
Montgomery et al. [17]	1995	7 (54)	6 (46)	0 (0)	10 (77)	3 (13)	0 (0)
Montgomery et al. [17]	1995	0 (0)	0 (0)	12 (0)	0 (0)	0 (0)	12 (100)
Shelbourne et al. [25]	2007	0 (0)	17 (100)	0 (0)	0 (0)	0 (0)	17 (100)
Owens et al. [18]	2007	28 (100)	0 (0)	0 (0)	28 (100)	0 (0)	0 (0)
Yeh et al. [34]	1999	0 (0)	0 (0)	25 (100)	0 (0)	25 (100)	0 (0)
Shapiro et al. [24]	1995	0 (0)	7 (100)	0 (0)	0 (0)	7 (100)	0 (0)
Harner et al. [7]	2004	1 (3)	29 (94)	1 (3)	1 (3)	28 (90)	2 (7)
Washer et al. [31]	1999	0 (0)	13 (100)	0 (0)	0 (0)	13 (100)	0 (0)
Scheffler et al. [22]	2009	10 (19)	29 (54)	14 (26)	14 (26)	25 (46)	14 (26)
Bin et al. [4]	2007	0 (0)	3 (20)	12 (80)	0 (0)	7 (47)	8 (53)
Thomsen et al. [29]	1984	4 (67)	0 (0)	2 (33)	4 (67)	0 (0)	2 (33)
Total		50 (23)	104 (47)	66 (30)	57 (26)	108 (49)	55 (25)

**Fig. 3:**
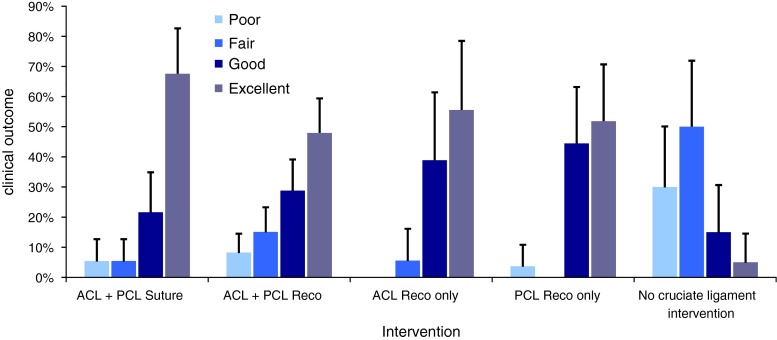
Mean values and standard deviations of grouped individual patient data are presented. Clinical results depending on *ACL* (anterior cruciate ligament) and *PCL* (posterior cruciate ligament) treatment. Ligament sutures (repair) and ligament reconstruction are compared with conservative treatment. The grades of *excellent*, *good*, *fair* and *poor* were pooled as results from the Lysholm and/or IKDC Score. No significant difference between suture repair and reconstruction could be detected. Operative treatment led to significantly better results than conservative therapy

Non-operative treatment of combined ACL and PCL ruptures (*n* = 27) resulted in moderate or poor outcomes in 70 % of the patients (Fig. 3). Forty patients undergoing suture repair of the ACL and PCL showed excellent and good results (40 and 37.5 %, respectively) (*p* < 0.001) compared with non-operative treatment (Fig. 3). ACL and PCL reconstruction (*n* = 73) led to excellent or good results (28 and 45.3 %, respectively) (*p* < 0.001) compared with non-operative treatment (Fig. 3). No significant difference in clinical outcome could be found when comparing ligament suture versus ACL and PCL reconstruction (n.s.).

**Fig. 4:**
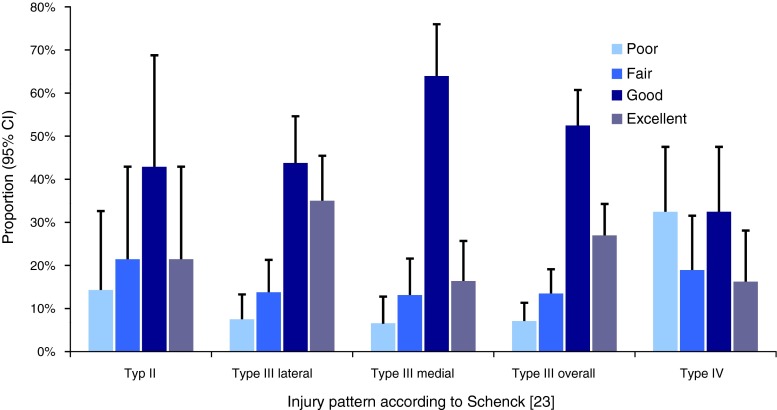
Mean values and standard deviations of grouped individual patient data are presented. Clinical results depending on the pattern of the injury according to Schenck’s classification. The grades of *excellent*, *good*, *fair* and *poor* were pooled as results from the Lysholm and/or IKDC Score. Schenck Type IV injuries had a significantly worse outcome than lesser knee dislocation types

With increasing injury severity according to Schenck’s classification, the clinical result became significantly worse (*p* = 0.0453, odds ratio, OR = 2.289 (1.018–5.150)) (Fig. 4). There was no statistically significant difference in the clinical outcome between type II and type III knee dislocations.

## Discussion

The most important finding of the presented study is that suture repair of cruciate ligaments leads to similar clinical results than cruciate ligament reconstruction in multiligament injuries of the knee. As shown in our study, good and excellent results were achieved by suture repair of the ACL and PCL in 77.5 % of patients with type III and IV knee dislocations, according to Schenck’s classification [23]. These clinical results are comparable to those accomplished by ligament reconstruction. A major problem of most published articles about knee dislocations is the lack of homogeneity in injury pattern, as well as the variety of different treatment regimens. As shown in our study the injury pattern according to Schenck has a significant influence on the clinical outcome. Former meta-analyses of knee dislocations did not consider the severity and pattern of the injury [3, 11]. To our knowledge, this study is the first meta-analysis of knee dislocations based on individual patient data with the possibility of matching patients to distinct injury patterns to obtain more homogeneous treatment groups.

This study confirms a previous study that reported poor outcomes after non-operative treatment for knee joint dislocation [8]. In contrast, suture of the LCL significantly improves clinical outcome compared with conservative treatment. Generally, posterolateral corner injuries should be surgically treated because delayed treatment is less successful [16]. It has also been shown that patients with multiligament injuries who undergo suture repair within 3 weeks after the trauma have a significantly better outcome compared to patients who undergo surgery more than 3 weeks after the injury [11]. Three weeks after the trauma is widely accepted as the threshold between ‘acute’ and ‘chronic’ [5, 7, 11, 12]. After 3 weeks, anatomical suture repair of ligaments is insufficient owing to scarring, retraction of ligament stumps, and granulation [5, 7, 11, 12]. Moreover, Richter et al. [20] showed significantly better results for ligamental suture repairs performed within 1 week of trauma compared with delayed repair (>1 week). Hence, suture repairs in multiligament injuries should be performed within 3 weeks if ligament suture within the first week is not achievable.

According to our data, operative treatment of acute knee dislocations should consist of suture repair or reconstruction of the cruciate ligaments. Good clinical results also have been described after delayed ligament reconstruction for knee joint dislocation [5, 9]. Karataglis et al. [9] studied 35 patients who received operative treatment (ligament reconstruction) at a mean of 32 months after injury. Sixty per cent of these patients reported excellent or good outcomes [9]. Fanelli and Edson [5] studied 41 PCL/PLC-injured patients who received treatment from 4 to 240 months after injury with good functional results at a minimum of 24 months follow-up. Nevertheless, owing to the poor clinical outcome after conservative therapy as shown in this study, we recommend the early surgical treatment (suture or reconstruction) of all injured ligaments in patients with a dislocated knee joint.

Arthroscopic cruciate ligament reconstructions in knee dislocations are not recommended within the first days after injury because of the possible development of compartment syndrome [19]. This complication should be closely considered in patients with high-energy traumas. Early ligament reconstruction has also been described as an additional risk factor for arthrofibrosis [2, 14, 15, 26]. There are no reports about the relation between arthrofibrosis and suture repair of the ACL, whereas a study has shown that the risk after a ligament reconstruction is greater 1.7 % [2]. Especially in knee joint dislocation, cruciate ligament reconstruction may be expected to lead to greater arthrofibrosis rates. The rate of arthrofibrosis after ligament suture versus ligament reconstruction in knee dislocations has not been examined until now.

Several studies have shown good and excellent results for primary ligament sutures [16, 18]. Owens et al. [18] found a mean Lysholm score of 89 points after a 48-month follow-up in 25 patients with knee joint dislocation who underwent primary repair of all damaged ligaments. Conversely, Mariani et al. [16] found no difference in functional outcomes when comparing primary suture repair versus reconstruction of combined ACL/PCL injuries but noted a greater degree of flexion loss and PCL instability and a lower rate of return to pre-injury activity levels in those who had primary suture repair of the cruciate ligaments. Stannard et al. [28] recently reported the results of a prospective trial that directly compared suture repair versus reconstruction of the PLC in 57 knees, 44 (77 %) of which had multiligament injury. The minimum follow-up was 24 months. The repair failure rate was 37 % compared with a reconstruction failure rate of 9 %. This finding has not been confirmed by other studies [11] and contrasts with our results, where only 19 % of the patients with operative treatment (86.8 % suture repair and 13.2 % reconstruction) of the posterolateral corner had a poor or fair result. In the interpretation of these controversial results, it should be considered that in both the above-mentioned studies [11, 28], a high percentage of patients with an intact ACL were included, whereas the present study admitted only patients with a torn ACL and PCL. It must also be considered that in the suture repair group in many studies, the PCL was sutured and the ACL was left untreated [11]. Considering that our study shows that the surgical treatment of the ACL and PCL has a significant effect, it seems critical to compare only PCL sutures with PCL and ACL reconstructions.

Previous studies described significantly more ruptures of the lateral collateral ligament close to the attachment compared with avulsions of the medial collateral ligament (84 vs. 46 %, respectively) [30]. This finding may be related to the significantly greater improvement after surgical repair of the lateral collateral ligament as shown in this study, whereas patients did not profit from surgical repair of the medial collateral ligament compared with non-operative treatment. No study has been performed to distinguish the preferred treatment options for different rupture locations, such as avulsions versus intraligamentous ruptures.

To avoid additional surgical morbidity and the increased surgical time associated with harvesting grafts, multiple authors have opted for allograft reconstructions [4, 7, 24, 31]. A significant clinical advantage of using allografts versus autografts in knee dislocations could not be found in the presented study. Harner et al. [7] observed only one graft failure that required a reoperation in a series of thirty-one patients who had a total of sixty allograft cruciate reconstructions. Other authors have reported that the use of allografts may decrease the risk of arthrofibrosis [31], which has not been proven up to now. Nevertheless, there is no evidence for the use of allograft versus autograft in knee dislocations.

According to our data, a planned 2-stage procedure including operative treatment with suture repair of the collateral ligaments without addressing the cruciate ligaments in the primary phase cannot be recommended.

A limitation of our study was the lack of an indication in most of the considered articles regarding the exact suture technique performed (intra-ligamental vs. transosseous). Additionally, suture materials are not consistent, and absorbable and non-absorbable sutures have been utilised. Further limitations were the heterogeneity of the patients themselves, the mechanisms of injury, low- versus high-energy trauma, the injury patterns, and their associated traumas, as well the issues of polytrauma and the unknown presence of ipsilateral fractures in the study population.

## Conclusion

Conservative treatment after knee dislocation yields poor clinical response. Suture repair of cruciate ligaments can still be an alternative treatment option for type III and IV knee dislocations, according to Schenck’s classification and can achieve good clinical results, which are comparable to that of ligament reconstructions. The injury pattern significantly influences the clinical outcome. The data provided by this meta-analysis should be supported with a future prospective study in which suture repair and ligament reconstruction are compared.
